# Advancing Care Team Adoption of Electronic Health Record Systems for Cancer Symptom Management: Findings From a Hybrid Type II, Cluster-Randomized, Stepped-Wedge Trial

**DOI:** 10.1200/OP.24.00280

**Published:** 2024-08-06

**Authors:** Jessica D. Austin, Lila J. Finney Rutten, Kristin Fischer, Jennifer Ridgeway, Sarah Minteer, Joan M. Griffin, Deirdre R. Pachman, Kathryn J. Ruddy, Andrea Cheville

**Affiliations:** ^1^ Division of Epidemiology, Quantitative Health Sciences, Mayo Clinic, Scottsdale, AZ; ^2^ Division of Epidemiology, Quantitative Health Sciences, Mayo Clinic, Rochester, MN; ^3^ Mayo Clinic Comprehensive Cancer Center, Rochester, MN; ^4^ Division of Health Care Delivery Research and Robert D. and Patricia E. Kern Center for the Science of Health Care Delivery, Mayo Clinic, Rochester, MN; ^5^ Division of Medical Rehabilitation, Physical Medicine and Rehabilitation, Mayo Clinic, Rochester, MN; ^6^ Division of Community Internal Medicine, Geriatrics, and Palliative Care, Mayo Clinic, Rochester, MN; ^7^ Division of Medical Oncology, Mayo Clinic, Rochester, MN

## Abstract

**PURPOSE:**

The enhanced, electronic health record (EHR)–facilitated cancer symptom control (E2C2) trial is a cohort cluster-randomized, stepped-wedge, hybrid type II trial that leverages EHR systems to facilitate a collaborative care model (CCM) approach with the goal of improving cancer symptom management. Understanding factors that influence care team adoption of EHR systems remains a critical understudied area of research. This study examines how oncology care teams' perceptions regarding the feasibility, acceptability, and appropriateness of E2C2 EHR systems preimplementation were associated with adoption 3 months after implementation and characterizes differences in adoption by individual- and system-level factors.

**METHODS:**

Care team members completed an electronic survey before and 3 months after implementation of E2C2 for their respective sequence. Adoption was defined as frequency of use to statements aligned with care team–directed EHR systems designed to facilitate CCM approaches. Chi-square tests assessed differences in adoption while logistic regression models estimated associations between baseline mean scores of acceptability, feasibility, and appropriateness on care team adoption at 3 months.

**RESULTS:**

Results from 94 care team members (37.2% oncologists, 72.6% female, 55.3% in their role for 6+ years) found that adoption rates ranged from 48.9% to 71.7%, with significant differences observed by location (community-based health care systems *v* tertiary medical center) and professional role. Adjusting for professional role, care team members reporting higher levels of perceived acceptability and appropriateness at baseline had greater odds of adopting EHR systems at 3 months.

**CONCLUSION:**

EHR systems perceived as acceptable and appropriate are more likely to be adopted by oncology care teams in our sample. Future implementation efforts should consider tailored strategies to facilitate adoption of EHR systems designed to promote CCM-based approaches to improve cancer symptom management.

## Teaser

Understanding adoption of EHR systems that promote collaborative care approaches to cancer symptom management.

## INTRODUCTION

Cancer and cancer treatment are associated with disabling symptoms that affect quality of life, adherence to recommended cancer treatments, health care utilization, financial toxicity, and survival.^1,2^ Despite the availability of effective, evidence-based care guidelines for symptom management for patients with cancer, the prevalence of uncontrolled symptoms remains unacceptably high.^3-5^ The increasing complexity of modern cancer care suggests that a team-based approach to the collaborative care model (CCM) may improve symptom management. However, CCM-based approaches have been challenging to scale and disseminate because of high human resource requirements, despite their exhaustive validation in diverse clinical contexts, including cancer symptom control.^6-9^

**CONTEXT**

**Key Objective**
Are electronic health record (EHR) systems designed to promote collaborative care model approaches to cancer symptom management adopted by oncology care teams and are oncology care teams perceptions of trial feasibility, acceptability, and appropriateness associated with adoption at 3 months after implementation?
**Knowledge Generated**
Results from 94 oncology care team members found that early adoption rates ranged from 48.9% to 71.7% with significant differences observed by location and professional role. Oncology care team members reporting higher levels of trial acceptability and appropriateness at baseline had greater odds of adopting EHR systems at 3 months. Feasibility was not associated with adoption.
**Relevance**
EHR systems perceived to be acceptable and appropriate by oncology care teams are more likely to be adopted in our sample, but adoption of such systems vary, emphasizing the need for implementation strategies tailored to the local context.

Leveraging electronic health records (EHRs) to facilitate core components of CCM-based approaches, including patient-reported outcome measure (PROM) collection, symptom-specific patient education, and clinician decision support, has considerable potential to neutralize barriers by lessening operational costs, and to improve management of symptoms in patients with cancer.

Randomized controlled trials have shown impressive benefits when electronic PROMs (ePROMs) are used for symptom surveillance and management in patients with cancer, including enhanced quality of life, reduction in hospital admissions, early detection of adverse events, reduction in emergency room visits, and increased patient survival.^10-13^ Despite these benefits, previous studies show that providing clinicians with symptom scores does not result in significant improvements in symptom management.^14-17^ These challenges have been attributed to time and resource constraints to review and coordinate symptom treatments. Parameterizing EHR systems to execute key components of CCM-based approaches offers potential to address these gaps and enable cancer care teams to be effective.

Implementation science is the study of methods and strategies that facilitate the uptake of innovations, interventions, or evidence-based interventions into practice.^18-20^ A landmark 2011 paper outlined a taxonomy of discrete implementation outcomes including acceptability, adoption, appropriateness, feasibility, fidelity, implementation cost, penetration, and sustainability.^21^ Implementation outcomes serve as indicators of implementation effectiveness and as necessary preconditions for attaining and sustaining subsequent desired changes in clinical outcomes.^21^ Adoption is conceptualized as a behavioral outcome and refers to the uptake or action to use an implementation target.^21-23^ Perceptual outcomes including acceptability, feasibility, and appropriateness are posited to be most salient to and predictive of intervention adoption, particularly in the formative and early phases of implementation.^21-23^ Acceptability is the perception that a given innovation is agreeable, palatable, or satisfactory. Appropriateness refers to the perceived fit, relevance, or compatibility of an innovation, while feasibility is the extent to which an innovation can be successfully used or carried out in a given setting.^21^ Measuring and understanding the relationship between implementation outcomes that capture perceptions and behaviors of key stakeholders is essential to understanding implementation processes and success—yet limited empirical evidence exists that assesses relationships between implementation outcomes at different points along the implementation process.^22,24^

The National Cancer Institute (NCI) funded the improving the management of symptoms during and after cancer treatment (IMPACT) Consortium, composed of three research centers and a coordinating center, to support the development, implementation, and evaluation of integrated electronic symptom management systems for patients with a history of cancer. Each center tested the use of EHR-embedded symptoms management interventions using ePROMs to assess and address common cancer symptoms.^25^ As one of three research centers participating in the IMPACT Consortium (UM1 CA 233033), the enhanced, EHR-facilitated cancer symptom control (E2C2) trial focused on six prevalent SPADE (sleep interference, pain, physical function limitations, anxiety, depression, and energy deficit/fatigue) symptoms and is based on a robustly validated CCM approach with patient- and care team–directed elements designed to increase the frequency with which patients receive individualized, preference-concordant, and guideline-based care for their symptoms. This paper aims to characterize care team adoption of oncology care team–directed EHR systems and to assess the association between acceptability, appropriateness, and feasibility on care team adoption.

## METHODS

### Study Design and Intervention Description

**FIG 1.**
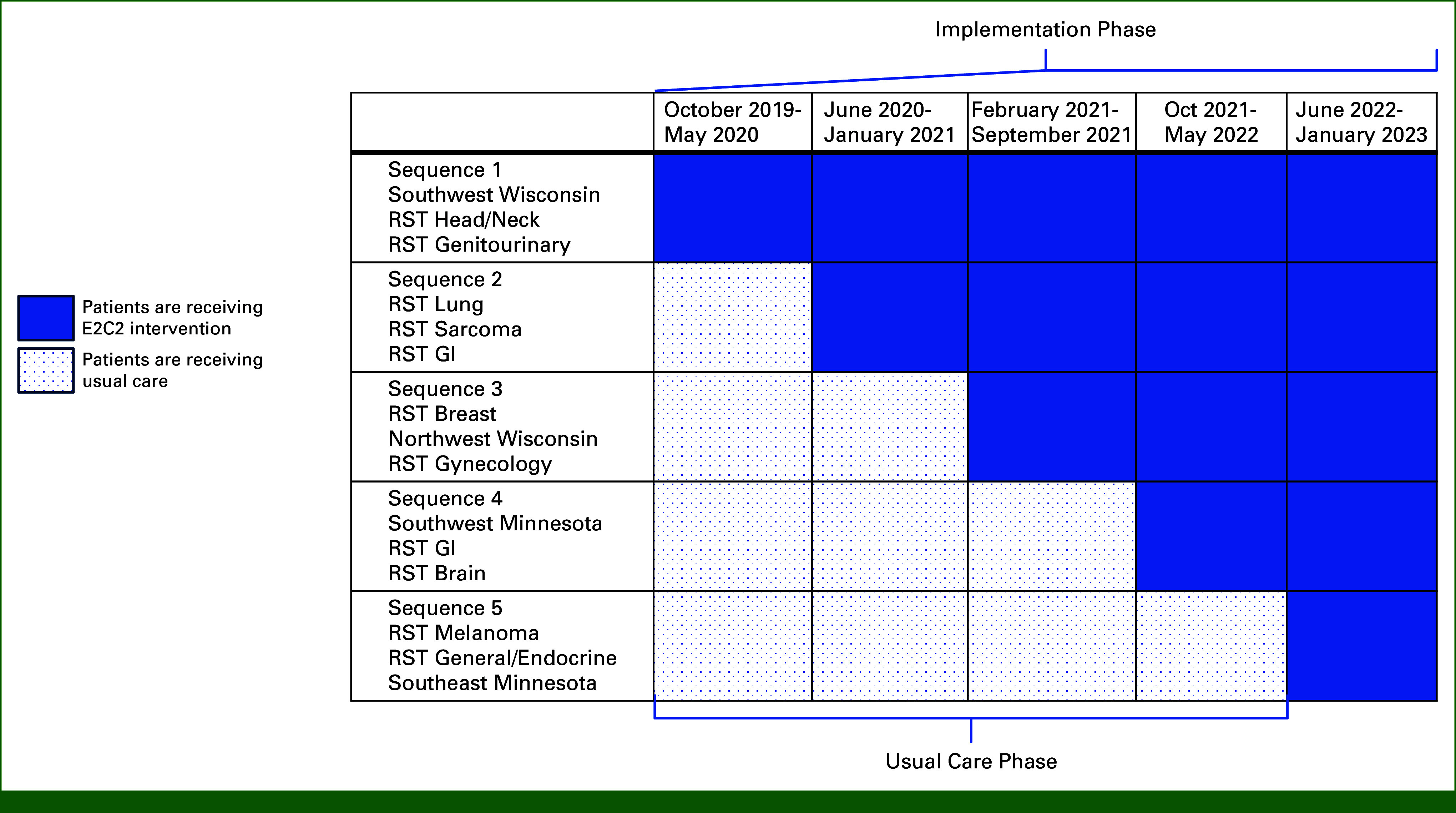
E2C2 stepped wedge design with 15 clusters (defined by site and cancer care team) randomly assigned to one of five sequences. E2C2, enhanced, electronic health record–facilitated cancer symptom control; RST, Rochester, MN.

The E2C2 study is a hybrid type 2, stepped-wedge, pragmatic, cohort cluster-randomized clinical trial aimed at evaluating the effectiveness and implementation of an EHR-enabled CCM-based approach to improve patient-reported symptom burden and functional status. The trial was conducted at Mayo Clinic in Rochester, MN, a tertiary NCI-designated comprehensive cancer center, and at Mayo Clinic Health System (MCHS) community hospitals and clinics in Minnesota and Wisconsin. In the stepped wedge design, 15 clusters (defined by site in the MCHS and cancer care team in Mayo Clinic, Rochester) were randomly assigned to one of five sequences. After a 6-month control-only period, sequences went live with the E2C2 intervention at 8-month intervals per the stepped wedge design (Fig 1). This study was approved and granted a waiver of informed consent by the Mayo Clinic Institutional Review Board (18-007779).

The E2C2 intervention targets patients and oncology care teams by deploying both a symptom control bundle and implementation bundle simultaneously. The symptom control bundle consisted of automated ePROM (patient-directed) data collection via the EHR and interactive voice response for SPADE symptoms, assessed from 0 to 10, with higher scores representing higher levels of symptom severity, and presentation of symptom data to patients and oncology care teams. Participants reporting moderate symptom severity (≥4/10) also received paper-based and automated delivery of SPADE self-management tool kits for moderate or worse symptoms, and participants reporting severe symptoms (≥7/10) received a referral to a registered nurse symptom care manager (RN SCM) (SCM; patient- and care team–directed). The implementation bundle deployed three evidence-based, multifaceted strategies designed to encourage care team adoption of the symptom control bundle: practice facilitation; best practice alerts (BPAs); and audit and feedback.^26^ This paper focuses on adoption of care team–directed EHR systems of the symptom and implementation bundle including BPAs, viewing patient symptom data in the EHR, and referrals to the SCM. Greater details on the trial are included in a previously published protocol paper.^27^

Our team deployed various strategies to inform care teams about the goal of E2C2 and the EHR functionalities before and during the early phases of implementation for each respective sequence. Study staff conducted in-person and virtual informational sessions to provide an overview of the study including which EHR systems interface with care teams (ie, RN SCM referrals, BPAs, and symptoms scores in the EHR). The study staff also conducted site visits to troubleshoot any workflow or technical issues at the sites before the interventions go live. Additionally, each care cluster was assigned a clinical champion who underwent training within the first month of implementation and served as local champions, internal facilitators, and relayed information between the study team and the sites.

### Data Collection and Measures

Clinical champions identified oncology care team members, including physician oncologists, advanced practice providers, nurses, and other staff (ie, desk/room staff, administration, operations, pharmacy, information technology, and other), were recruited to complete an electronic survey before implementation (baseline) and 3 months after the interventions go live for their respective sequence. The baseline and 3-month care team surveys included items assessing individual characteristics, as well as perceptions of the feasibility, acceptability, and appropriateness of the E2C2 intervention.^28^ Items measuring care team adoption were additionally included in the 3-month survey. The baseline survey was administered to 208 care team members (response rate 65%) and the 3-month surveys were administered to 203 care team members (response rate 62%). Two follow-up reminders were sent to those who did not complete the survey.

#### Primary Outcome

Care team adoption of the E2C2 intervention components was measured at 3 months after implementation for each respective sequence and measured as frequency of use in response to three statements that align with care team–directed EHR systems within the symptom and implementation bundles: (1) “I review patient symptom scores in my note, Snapshot, or Synopsis during encounters,” (2) “I open Best Practice Advisories triggered by severe symptom scores,” and (3) “I refer patients to or discuss patients with RN SCMs.”^27^ Each item was assessed on a four-point Likert scale (never, once, occasionally, and often) and dichotomized as never/once versus occasionally/often and aggregated at the sequence level. Care team members completing the survey had the option to select not relevant to my role to any item on the survey. To account for differences in care team workflows, roles, and responsibilities, care team members selecting not relevant to my role to an adoption item were excluded from analyses for that respective item.

#### Independent Variables

Care team members assessed their perceptions of acceptability, feasibility, and appropriateness of E2C2 using the validated and widely used 12-item Acceptability of Intervention Measure, Intervention Appropriateness Measure, and Feasibility of Intervention Measure (AIM-IAM-FIM) scales.^28^ These four-item scales assessed care team perceptions of the E2C2 intervention in the domains of satisfaction and agreeability (acceptability), perceived fit and relevance (appropriateness), and the degree to which it can be successfully operationalized (feasibility) with ordinal response options ranging from 1 = completely disagree to 5 = completely agree.^28^ A mean score for each scale was generated by averaging responses of the four items, with higher mean scores indicating higher levels of acceptability, appropriateness, and feasibility.

### Analysis

This analysis is limited to care team members responding to both the preimplementation and 3-month surveys. Respondents selecting not relevant to my role to one of three adoption items were also excluded from this analysis. The final analytic sample includes 94 care team members. Descriptive statistics were calculated to characterize care team members in terms of biologic sex, professional role, and years of experience in current role. We also summarized mean scores for care teams' baseline perceptions of the acceptability, appropriateness, and feasibility of the E2C2 intervention (AIM-IAM-FIM). Chi-square tests were used to examine associations between differences in adoption by professional role, biologic sex, age, length of time in role, study sequence, and cluster location (community-based health care system *v* tertiary medical center). Logistic regression models were used to examine the association between baseline measures of AIM, FIM, and IAM on care team adoption of E2C2 components at 3 months. All analyses were performed using IBM SPSS Statistics 28.

## RESULTS

Care team characteristics are summarized in Table 1. The majority were located at the tertiary medical center (58.5%), 37.2% were oncologists, 72.6% identified as female, and 55.3% had been in their role for at least 6 years. At 3 months after implementation, 71.7% of care team members reported occasionally or often reviewing patient symptoms scores in their notes, Snapshot, or Synopsis during encounters, 48.9% reported opening Best Practice Advisories triggered by severe symptom scores, and 56% reported referring or discussing patients to RN SCMs.

**TABLE 1.** Summary of Care Team Characteristics (No. = 94)

Characteristic	No. (%)
Sequence	
1	16 (17.0)
2	22 (23.4)
3	30 (31.9)
4	15 (16.0)
5	11 (11.7)
Location	
Community-based health system	39 (41.5)
Tertiary medical center	55 (58.5)
Role in cancer care delivery	
Physician oncologist	35 (37.2)
Advanced practice provider	16 (17.0)
Nurse	38 (40.4)
Other^a^	5 (5.3)
Sex	
Male	25 (72.2)
Female	65 (72.2)
Length of time in role	
Less than 1 year	10 (10.6)
1-2 years	14 (14.9)
3-5 years	18 (19.1)
6-10 years	26 (27.7)
11-15 years	9 (9.6)
More than 15 years	17 (18.1)

^a^ Other includes any care team member identifying as desk/room staff, administration, operations, pharmacy, information technology, or other.

We examined differences in adoption of E2C2 intervention components at 3 months after implementation by care team characteristics (Table 2). On the basis of responses to the item, “I review patient symptom scores in my note, Snapshot, or Synopsis during encounters,” significant differences in care team adoption were observed by location and professional role, with team members located in the community-based health care system, nurses, and other staff (ie, desk/room staff, administration, operations, pharmacy, information technology, and other) reporting significantly lower rates of adoption. Significant differences by professional role were also observed regarding adoption of Best Practice Advisories triggered by severe symptom scores (≥7/10 for any one symptom); physician oncologists and advanced practice providers reported higher rates of adoption compared with nurses and other staff (ie, desk/room staff, administration, operations, pharmacy, information technology, and other). We did not observe any significant differences in adoption for referring or discussing patients with RN SCMs by care team or study characteristics.

**TABLE 2.** Differences in Care Team Adoption at 3 Months by Care Team Characteristics

Variable	I Review Patient Symptom Scores in My Note, Snapshot, or Synopsis During Encounters (No. = 92)		I Open Best Practice Advisories Triggered by Severe Symptom Scores (No. = 90)		I Refer Patients to or Discuss Patients With RN Symptom Care Managers (No. = 91)	
	No. (%)^a^	*P*	No. (%)^a^	*P*	No. (%)^a^	*P*
Adoption rate	66 (71.7)		44 (48.9)		51 (56.0)	
Sequence		.28		.10		.70
1	10 (15.1)		3 (6.8)		9 (17.6)	
2	18 (27.3)		12 (27.3)		11 (21.6)	
3	22 (33.3)		18 (40.9)		19 (37.2)	
4	11 (16.7)		6 (13.6)		7 (13.7)	
5	5 (7.6)		5 (11.4)		5 (9.8)	
Location		.02		.50		.57
Community-based health system	22 (66.7)		17 (38.6)		20 (39.2)	
Tertiary medical center	44 (33.3)		27 (61.4)		31 (60.8)	
Role in cancer care delivery						
Physician oncologist	28 (42.4)	.02	21 (47.7)	.04	23 (45.1)	.24
Advanced practice provider	14 (21.2)		10 (22.7)		9 (17.6)	
Nurse	22 (33.3)		12 (27.3)		18 (35.3)	
Other^a^	2 (3)		1 (2.3)		1 (2)	
Sex		.43		.08		.22
Male	20 (31.2)		15 (36.6)		16 (32.6)	
Female	44 (68.7)		26 (63.4)		33 (67.3)	
Length of time in role		.11		.05		.66
Less than 1 year	5 (7.6)		4 (9.1)		4 (7.8)	
1-2 years	10 (15.1)		5 (11.4)		7 (13.7)	
3-5 years	10 (15.1)		10 (22.7)		10 (19.6)	
6-10 years	18 (27.3)		8 (18.2)		13 (25.5)	
11-15 years	8 (12.1)		4 (9.1)		5 (9.8)	
More than 15 years	15 (22.7)		13 (29.5)		12 (23.5)	

NOTE. Frequencies not adding up to total sample size indicate differences in relevance of each adoption item to the participants' role (ie, participants selecting not relevant to my role were excluded from analyses for respective adoption item).

Abbreviation: RN, registered nurse.

^a^ Column percent of the subset classified as adopting item.

**FIG 2.**
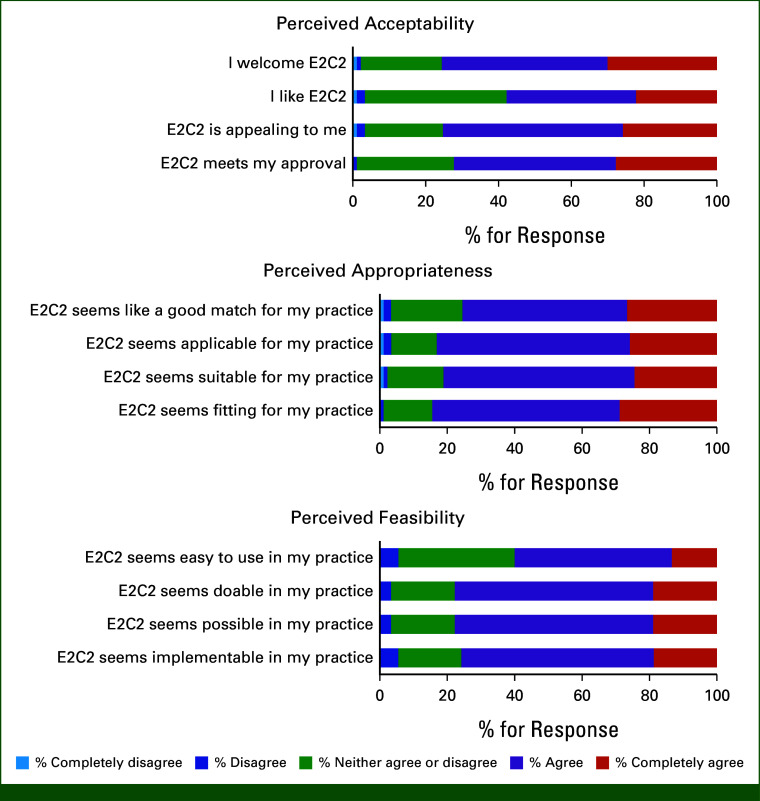
Distribution of oncology care team members responses to individual acceptability, appropriateness, and feasibility items at baseline. E2C2, enhanced, electronic health record–facilitated cancer symptom control.

Figure 2 shows that care teams indicated high levels of agreement to individual items comprising scale measures of acceptability (overall mean score, 3.9; standard deviation [SD], 0.74), appropriateness (overall mean score, 4.0; SD, 0.71), and feasibility (overall mean score, 3.9; SD, 0.7) of E2C2 at baseline. We did not observe significant differences in baseline means scores of acceptability, feasibility, or appropriateness by oncology care team characteristics (Appendix Table A1, online only). In unadjusted analyses (Table 3), there was a positive significant association between baseline perceived acceptability and all three measures of adoption at 3 months after implementation. Specifically, care teams members who reported higher levels of perceived acceptability of the E2C2 intervention had greater odds of reviewing patient symptom scores (odds ratio [OR], 2.1 [95% CI, 1.06 to 4.23]; *P* = .03), opening Best Practice Advisories (OR, 2.1 [95% CI, 1.2 to 4.0]; *P* = .01), and referring patients to or discussing patients with RN SCMs (OR, 2.3 [95% CI, 1.2 to 4.4]; *P* = .01). Baseline levels of perceived appropriateness was borderline associated with greater adoption of reviewing patient symptoms scores (OR, 2.0 [95% CI, 1.0 to 4.18]; *P* = .05) and borderline associated with referring patients to and discussing patients with RN SCMs (OR, 1.9 [95% CI, 1.0 to 3.7]; *P* = .05).

**TABLE 3.** Unadjusted Logistic Regression Models Examining the Association Between Baseline Implementation Outcomes and Adoption of Intervention Components at 3 Months After Implementation for All Sequences

Variable	I Review Patient Symptom Scores in My Note, Snapshot, or Synopsis During Encounters		I Open Best Practice Advisories Triggered by Severe Symptom Scores		I Refer Patients to or Discuss Patients With RN Symptom Care Managers	
	OR (95% CI)	*P*	OR (95% CI)	*P*	OR (95% CI)	*P*
Acceptability	2.1 (1.06 to 4.23)	.03	2.1 (1.2 to 4.0)	.01	2.3 (1.2 to 4.4)	.01
Feasibility	1.1 (0.54 to 2.14)	.83	1.6 (0.8 to 2.9)	.15	1.6 (0.9 to 3.0)	.12
Appropriateness	2.0 (1.0 to 4.18)	.05	1.8 (0.98 to 3.5)	.06	1.9 (1.0 to 3.7)	.05

Abbreviations: OR, odds ratio; RN, registered nurse.

Adjusting for professional role (Table 4), preimplementation acceptability remained significantly associated with all three measures of care team adoption at 3 months after implementation, and the strength of the associations increased. Appropriateness was also found to be significantly associated with all three measures of care team adoption and the strength of the association increased. Baseline perceived feasibility scores were not associated with care team adoption at 3 months after implementation in unadjusted or adjusted analyses.

**TABLE 4.** Logistic Regression Models Adjusted for Role in Care Delivery Examining the Association Between Baseline Implementation Outcomes and Adoption of Intervention Components at 3 Months After Implementation for All Sequences

Variable	I Review Patient Symptom Scores in My Note, Snapshot, or Synopsis During Encounters		I Open Best Practice Advisories Triggered by Severe Symptom Scores		I Refer Patients to or Discuss Patients With RN Symptom Care Managers	
	OR (95% CI)	*P*	OR (95% CI)	*P*	OR (95% CI)	*P*
Acceptability	2.6 (1.2 to 5.7)	.02	2.4 (1.3 to 4.8)	.01	2.4 (1.2 to 4.9)	.01
Feasibility	1.2 (0.5 to 2.6)	.65	1.8 (0.9 to 3.5)	.08	1.6 (0.8 to 3.2)	.11
Appropriateness	2.5 (1.1 to 5.5)	.02	2.1 (1.1 to 4.1)	.03	2.0 (1.0 to 4.0)	.03

Abbreviations: OR, odds ratio; RN, registered nurse.

## DISCUSSION

Parameterizing EHRs to systematize and promote CCM-based approaches for cancer symptom management holds tremendous promise if appropriately implemented and adopted by care teams. Moreover, understanding factors that influence care team adoption can generate important insights to guide efforts to improve implementation, scaling, and dissemination. Overall, we found that care team adoption of E2C2 intervention components varied by professional role (with less adoption by nurses and other care team members than by physicians and advanced practice providers) and location (with less adoption reported by community-based care teams than by care teams in the tertiary medical center). Additionally, we observed that baseline levels of acceptability and appropriateness were significantly associated with all three measures of care team adoption at 3 months after adjustment for professional role. We did not observe any significant associations between care teams perceptions of E2C2 feasibility and subsequent care team adoption.

Care teams' perceptions of intervention feasibility, acceptability, and appropriateness are hypothesized to be salient to intervention adoption, particularly during the early phases of implementation,^21,29^ but these relationships remain largely understudied in the literature.^22^ Although higher levels of preimplementation acceptability and appropriateness were significantly associated with greater rates of care team adoption of our symptom and implementation bundles, adjusting for professional role, preimplementation feasibility was not found to be associated with adoption in our population. It is possible that measurement limitations contributed to this finding. Challenges in the measurement and performance of implementation outcome measures are well described^30-32^ and could make it difficult to detect effects. Although psychometrically evaluated, the measures used to assess feasibility, acceptability, and appropriateness are relatively new, semantically similar, lack discriminative validity, and have not been applied to clinical care teams in the context of cancer symptom management or EHR systems.^28^ Moreover, few, if any, studies have assessed the predictive validity of the measures on adoption.^22,28^ Difficulties distinguishing these constructs empirically could mean that measures of acceptability and appropriateness served as proxies of feasibility. The items assessing these constructs also referred to the intervention more broadly as opposed to the specific EHR-related activities performed by care teams that were included in the 3-month assessment (ie, E2C2 meets my approval, rather than reviewing symptom scores during patient encounters meets my approval). Thus, there is a need for broader efforts to improve the measurement of implementation outcomes and additional research exploring the role of preimplementation perceptions of feasibility, acceptability, and appropriateness, on adoption.

Adoption of a practice innovation involves a complex interplay of changes that occur over time and can vary by setting. Approximately half of the respondents self-reported adopting two of three EHR components directed at care teams. This finding is likely an overestimation of actual adoption rates. The survey was administered to care team members identified by clinical champions that completed both the baseline and 3-month surveys and may include perspectives from members more aware of E2C2 overall. Additionally, variable rates of adoption may also reflect differences in role responsibilities across practices and frequency of interaction with the EHR systems. For instance, the BPAs were only viewable upon entering a patient encounter note in the EHR. Although available to any care team member upon entering a patient's encounter note, this likely explains higher rates of adoption observed among physician oncologists and advanced practice providers. It is also possible that nurses and other staff rated their adoption as low to items that they should have selected as not relevant to my role. Care team members based in the health care system reported lower levels of adoption across all measures, suggesting the potential need for additional strategies related to engagement and implementation planning. Readiness to change is considered a precursor to successful implementation of complex changes in health care settings.^33^ Resistance to change is not uncommon if individuals and organizations perceive a change as unnecessary, unjustified, or driven by top-down political forces^33,34^; such perceptions result in lower levels of adoption. It follows that different strategies may be needed depending on differences in capacity, characteristics, resources, and organizational behavior.^35^ Future research should explore the relationship between readiness and preimplementation outcomes, and assess the impact on subsequent adoption.

Although this study addresses critical gaps in research, it is not without limitations. Data were collected from a single, high-resourced health care enterprise, limiting our ability to generalize to other, particularly lower-resourced settings. However, care team members included in this analysis are relatively diverse, allowing our team to understand differences in implementation outcomes by multilevel factors. All implementation outcomes included in this analysis were collected via self-report from care team members identified by clinical champions. Thus, reported levels of feasibility, acceptability, and appropriateness may reflect selection bias and overestimate these parameters. Our analysis excluded care team members who did not respond to at least one adoption item at 3 months or who selected not relevant to my role. Although this approach improved our data quality, it decreased our sample size, which may limit our ability to detect significant differences. Sample size limitations also hindered our ability to examine differences at the cluster level, where differences in patient volume and acuity on the basis of tumor type and severity likely exist. Our findings also suggest that readiness to change, particularly motivation, may have contributed to our findings, but objective measures of organizational readiness were not included in baseline assessments.

In conclusion, this study provides critical insights for advancing adoption of EHR systems to promote CCM-based approaches for cancer symptom management, as well as for advancing the science of implementation research. Although this study supports that EHR systems perceived to be acceptable and appropriate to oncology care teams are more likely to be adopted, we observed variation in adoption rates across the function of the EHR element, location, and professional role. To this end, future implementation efforts should consider engaging care team members beyond informational sessions by tailoring strategies to local needs and organizational capacity, such as developing implementation plans or recruiting staff to support workflow redesigns. Building EHR systems and tailoring strategies to promote CCM-based approaches at both the patient and clinician levels can be costly and time-consuming. Thus, more research is needed to study which strategies are most effective at improving adoption, a key indicator of implementation success. Specifically, future research endeavors should also examine the relative importance and relationship between clinician adoption of EHR systems on symptom burden among patients with cancer. This information is critical for understanding underlying mechanisms of change, which will inform widespread implementation efforts by guiding decisions on where to invest time and resources.

## Data Availability

The data sets used and/or analyzed during the current study are available from the corresponding author on reasonable request.

## APPENDIX

**TABLE A1.** Differences in Baseline Acceptability, Feasibility, and Appropriateness by Oncology Care Team Characteristics

Variable	AIM, Mean (SD)		FIM, Mean (SD)		IAM, Mean (SD)	
Overall	3.9 (0.7)		3.9 (0.7)		4.0 (0.7)	
Sequence		0.98		0.39		0.91
1	3.9 (0.8)		3.6 (0.7)		3.9 (0.7)	
2	3.9 (0.8)		3.8 (0.7)		4.1 (0.8)	
3	4.0 (0.7)		4.0 (0.7)		4.0 (0.7)	
4	3.9 (0.8)		3.7 (0.9)		4.1 (0.7)	
5	4.0 (0.6)		4.0 (0.6)		4.1 (0.5)	
Location		0.84		0.85		0.78
Community-based health system	3.9 (0.8)		3.9 (0.7)		4.1 (0.8)	
Tertiary medical center	3.9 (0.7)		3.8 (0.6)		4.0 (0.6)	
Role in cancer care delivery		0.24		0.11		0.71
Physician oncologist	4.0 (0.8)		4.0 (0.7)		4.1 (0.8)	
Advanced practice provider	3.7 (0.8)		3.5 (0.8)		3.9 (0.8)	
Nurse	3.9 (0.6)		3.9 (0.6)		4.1 (0.5)	
Other^a^	4.4 (0.4)		4.2 (0.3)		4.2 (0.5)	
Sex		0.64		0.11		0.79
Male	4.0 (0.8)		4.1 (0.7)		4.0 (0.6)	
Female	3.9 (0.7)		3.8 (0.7)		4.0 (0.8)	
Length of time in role		0.42		0.76		0.88
Less than 1 year	3.8 (0.6)		4.0 (0.3)		4.1 (0.4)	
1-2 years	3.7 (0.6)		3.7 (0.7)		3.9 (0.6)	
3-5 years	3.9 (0.6)		3.9 (0.6)		4.0 (0.6)	
6-10 years	4.0 (0.9)		3.9 (0.8)		4.0 (0.9)	
11-15 years	3.6 (0.7)		3.6 (1.0)		4.1 (0.9)	
More than 15 years	4.2 (0.7)		3.9 (0.6)		4.2 (0.7)	

Abbreviations: AIM, Acceptability of Intervention Measure; FIM, Feasibility of Intervention Measure; IAM, Intervention Appropriateness Measure; SD, standard deviation.

^a^ Other includes any care team member identifying as desk/room staff, administration, operations, pharmacy, information technology, or other.
